# Brazilian Subjective Cognitive Decline (BRASCODE) Cohort: baseline sociodemographic and clinical data

**DOI:** 10.1002/alz.091401

**Published:** 2025-01-09

**Authors:** Ana Letícia Amorim de Albuquerque, Manuella Edler Zandoná Giordani, Victória Tizeli Souza, Simone Sieben da Mota, Bruno De Olive De Marchi, Gabriela Raquel Paz Rivas, Guilherme Da Silva Carvalho, Haniel Bispo De Souza, Lucas Bastos Beltrami, Rhaná Carolina Santos, Wyllians Vendramini Borelli, Giovanna Carello‐Collar, Marcia L Fagundes Chaves, Eduardo R. Zimmer

**Affiliations:** ^1^ Universidade Federal do Rio Grande do Sul, Porto Alegre Brazil; ^2^ Hospital de Clínicas de Porto Alegre, Porto Alegre, Rio Grande do Sul Brazil; ^3^ Universidade Federal do Rio Grande do Sul, Porto Alegre, RS Brazil; ^4^ Universidade do Vale do Rio dos Sinos, São Leopoldo, Rio Grande do Sul Brazil; ^5^ Federal University of Rio Grande do Sul, Porto Alegre, Rio Grande do Sul Brazil; ^6^ Clinical Hospital of Porto Alegre, Porto Alegre, Rio Grande do Sul Brazil; ^7^ Federal University of Rio Grande do Sul (UFRGS), Porto Alegre, RS Brazil

## Abstract

**Background:**

Subjective cognitive decline (SCD) is the self‐perceived impairment of cognitive function and has been studied as a risk factor for objective cognitive impairment. Monitoring patients with SCD can aid in understanding the risk factors for dementia in low‐ and middle‐income countries. We aimed to describe the baseline sociodemographic and clinical data of the Brazilian Subjective Cognitive Decline (BRASCODE)

**Methods:**

BRASCODE is an observational, prospective cohort study of cognitively unimpaired individuals with > 65 years‐old with cognitive complaints conducted at the Hospital de Clínicas de Porto Alegre, Brazil. The baseline evaluation was performed from March 2022 to January 2024. Exclusion criteria were previous diagnosis of dementia or cerebrovascular disease and uncontrolled neuropsychiatric/clinical illness. Sociodemographic, memory complaint scale (MCS), Mini‐Mental State Examination (MMSE), Clinical Dementia Rating (CDR) Scale and Cognitive Reserve Scale (CRS) were collected. Blood, cerebrospinal fluid, and imaging Alzheimer’s disease (AD) biomarkers were also performed.

**Results:**

A total of 144 patients were included, with 72.2% being female. The majority were of white ethnicity (92.1%), belonging to middle‐class socioeconomic status (B and C ‐ 79.9%). The median age was 69 years, and the median education level was 15 years. The most prevalent comorbidities were arterial hypertension (46.5%), diabetes mellitus (14.6%), and smoking (6.9%). The median score on the MMSE was 29 [27, 30] points. Regarding the CDR scale, 109 patients (75.7%) had a CDR of 0, and 35 (24.3%) had a CDR of 0.5. CRS scores exhibited a positive correlation with MMSE (rho = 0.304; p = 0.0002) and years of formal education (rho = 0.408; p<0.0001). Age correlated negatively with MMSE (rho = ‐0.188; p = 0.024) and MCS (rho = ‐0.245; p = 0.003). MCS score of the subject and informant also correlated (rho = 0.305; p = 0.0002) and exhibited no correlation with CRS scores and MMSE.

**Conclusions:**

Our results show an association between the patient’s cognitive reserve and education and formal cognitive testing (MMSE). The future availability of AD biomarkers and follow‐up data will provide additional information about cognitive progression of the SCD participants.

